# The Oxylipin Dependent Quorum Sensing System enhances Pseudomonas aeruginosa dissemination during burn-associated infection

**DOI:** 10.21203/rs.3.rs-5073300/v1

**Published:** 2024-09-25

**Authors:** Eriel Martinez, Hansol Im, Javier Campos-Gomez, Carlos J. Orihuela

**Affiliations:** Heersink School of Medicine, The University of Alabama at Birmingham, Birmingham, Alabama, United States of America; Heersink School of Medicine, The University of Alabama at Birmingham, Birmingham, Alabama, United States of America; Cystic Fibrosis Research Center; Heersink School of Medicine, The University of Alabama at Birmingham, Birmingham, Alabama, United States of America

## Abstract

Following severe burn injury, *Pseudomonas aeruginosa* is the leading cause of life-threatening infection. Herein, we unveil how *P. aeruginosa* strategically employs host-derived oleic acid, released as consequence of burn-injury, to induce a hypervirulent phenotype via its Oxylipin Dependent Quorum Sensing system (ODS). ODS activation enhanced *P. aeruginosa* invasion of burned skin and promoted its dissemination to distant organs in vivo. ODS regulation of *P. aeruginosa* virulence involved the control of nitic oxide levels, a key signaling molecule in bacteria, through upregulation of the nitric oxide reductases NorCB. Immunization with OdsA, one of the enzymes involved in oxylipin generation, or treatment with a pharmacological inhibitor of OdsA, protected mice against lethal *P. aeruginosa* infection following burn-injury. Our findings reveal a new mechanism underlying *P. aeruginosa* hypervirulence in burn wounds and identifies OdsA as a promising target for preventing disseminated infections following burns.

## Introduction

*Pseudomonas aeruginosa* is able to cause acute and chronic infections as result of its ability to sense and rapidly adapt to new or altered environmental conditions ^1^. However, our understanding of the host-specific molecular cues and physiological triggers responsible for its transition between aggressive and persistent phenotypes remains incomplete ^2^. Recently, we described a new mechanism that allows *P. aeruginosa* to recognize the host and activate a specific genetic program that strongly affects bacterial physiology ^3^. This system, called the Oxylipin-Dependent quorum sensing System (ODS), employs host-derived oleic acid as the precursor for the enzymatic synthesis of oxylipins, specifically (10*S*)-Hydroxy-(8*E*)-octadecenoic acid (10-HOME) and (7*S*,10*S*)-hydroxy-(8*E*)-octadecenoic acid (7,10-DiHOME) ^4^. In controlled *in vitro* settings, 7-HOME and 7,10-DiHOME generated by the ODS autoinducer synthase, OdsA, accumulate in the extracellular milieu following the addition of oleic acid ^5^. These molecules, in turn, are imported into the bacterial cytoplasm and induce the expression of the ODS regulon, which controls twitching motility and biofilm formation ^3,6^. Our earlier work demonstrated the contribution of ODS to *P. aeruginosa* virulence in plants and insects. The current study expands on this and reveals ODS’s parallel significance in mammals.

Oleic acid is the predominant fatty acid in human adipose tissue and a major constituent across various tissues ^7^. While the majority of oleic acid in healthy tissues is esterified with glycerol forming triglycerides, studies have shown a dramatic increase in the amount of free oleic acid in tissues and plasma of patients with severe burn injuries ^8^. Interestingly, the pathogenesis of *P. aeruginosa* during infection of open wounds, in particular burn lesions, is considerably different from the less invasive yet persistent phenotype observed during other type of infections such as that occurring in the lung of cystic fibrosis patients where access to adipose tissue is limited ^9,10^. One key difference being that burn-related *P. aeruginosa* infections are marked by rapid proliferation and bloodstream dissemination to distant organs, contributing significantly to morbidity and mortality of burn patients worldwide ^11^. However, the underlying molecular drivers behind *P. aeruginosa’s* hypervirulence when in a burn injury setting remained unknown.

The clinical observation of elevated oleic acid in tissues following burn injury, together with our previous results on the role of the ODS on *P. aeruginosa* pathogenesis, prompted us to explore a potential role for ODS in this context. Our results show ODS has an important role during mammalian infection, establishing it as a key regulator of virulence among those with infected burns, and demonstrate its potential as a target for intervention. Our findings therefore advance our understanding of *P. aeruginosa* pathogenesis and provide a molecular explanation for its hyper-virulence among individuals with infected burns.

## Results

### ODS promotes *P. aeruginosa* skin colonization and dissemination in a mouse burn model.

**Figure 1.**
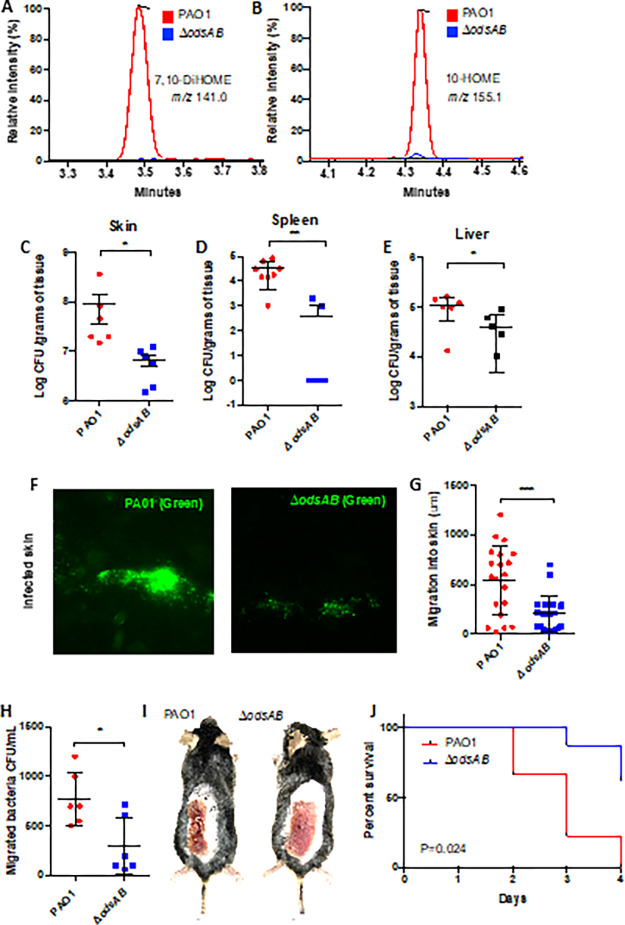
ODS promotes disseminated *P. aeruginosa* infection from a burn lesion. LC/MS/MS spectrometry analysis of homogenized skin from burned mice infected with PAO1 or *ΔodsAB*. Reconstructed ion chromatograms demonstrate the presence of A) 7,10-DiHOME (m/z 297.3) and B) 10-HOME (m/z 155.1) exclusively in mice infected with PAO1 (depicted in red), while absent in those inoculated with *ΔodsAB*(depicted in blue). Additionally, burned mice infected with *ΔodsAB*exhibited C) reduced skin colonization compared to WT PAO1, along with impaired dissemination to both the D) spleen and E) liver. Burn mice were infected with either PAO1 (PAO1-*gfp*) or *ΔodsAB* expressing GFP (*ΔodsAB-gfp*). Subsequent fluorescent microscopy analysis showed F) limited *ΔodsAB*colonization of the skin tissue compared to WT PAO1 (Panel B). G) Quantitative analysis revealed deeper migration of PAO1 into the skin compared to *ΔodsAB*. Moreover, results from H) an *in vitro* trans-well assay measuring the ability of an equal number of bacteria to cross a confluent monolayer of vascular endothelial cells. Notably, I) burned mice infected with PAO1 displayed hindered skin healing relative to those infected with *ΔodsAB*. Likewise, J) Kaplan Meier survival curves revealed a significant reduction in *ΔodsAB*infection-associated mortality compared to PAO1. Statistical analyses was performed using a two-tailed Student’s t-test with asterisks denoting significance levels (* for P < 0.05, ** for P < 0.01, and *** for P < 0.001). Mantel-Cox test was employed for survival data in Panel H. Shown are the combined results from at least two experiments, with each dot representing a biological replicate.

*P. aeruginosa* is highly aggressive when infecting burn sites; diverging from the chronic infections caused by *P. aeruginosa* in the airway ^10^. Oleic acid is also known to be present within burned tissues at high concentration ^8^. Building upon these observations we hypothesized that oleic acid present in the burn microenvironment may serve as a precursor for the synthesis of oxylipins activating ODS and that ODS may play an especially important role during burn-related *P. aeruginosa* infections. To test this hypothesis, the skin of three infected mice and three uninfected controls, both previously burned using a heated aluminum block ^12^, were isolated and homogenized. Subsequent to organic extraction, the samples underwent analysis via mass spectrometry. This analysis confirmed the presence of the ODS autoinducers 10-HOME and 7,10-DiHOME in the skin of mice infected with wildtype PAO1, and as expected, and not in those from mice infected with an in-frame deletion mutant of *odsAB* (*ΔodsAB*), i.e., lacking the genes responsible for the conversion of oleic acid to oxylipins (Fig. 1A and B respectively). We next tested whether ODS activation in the context of *P. aeruginosa*-infected burns contributed to skin colonization and bacterial dissemination to internal organs. We first observed that *P. aeruginosa* struggles to established itself within healthy skin and does not disseminate (Fig S1). This was in stark contrast to infections in mice that had experienced burn injury. Under burn conditions, both PAO1 and *ΔodsAB* were able to colonize the injured skin when infected intradermally with 10^5 CFU, however, mice infected with *ΔodsAB* displayed ~ 10-fold reduced bacterial burden when compared to the WT strain (Fig. 1C). Moreover, our experiments revealed that *ΔodsAB* has starkly diminished dissemination from the skin to the spleen (Fig. 1D) and liver (Fig. 1E). Further examination of infected skin sections by fluorescence microscopy, using PAO1-*gfp* or *ΔodsAB-gfp*, confirmed that *ΔodsAB-gfp* exhibited a reduced capacity to establish a skin infection (Fig. 1F) and suggested this correlated with restricted migration into the deeper layers of the skin. This difference was confirmed in quantitative analyses of skin sections taken from infected mice (Fig. 1G). Importantly, *in vitro* trans-well experiments testing the ability of an equal number of bacteria to cross a confluent monolayer of endothelial cells, demonstrated the requirement for both oleic acid and *odsAB* in efficient translocation (Fig. 1H, Fig. S2). Our studies further revealed a consistent delay in the healing process of the burn lesions in mice infected with WT PAO1 compared to those infected with *ΔodsAB* (Fig. 1I). Mortality outcomes further emphasized the importance of ODS in mammalian burn wound infections. While PAO1 parental strain resulted in 100% mortality within 48 hours post-burn/infection, *ΔodsAB* mutants induced only half of the mortality rate at the same time point (Fig. 1J). In summary, our findings highlight the critical involvement of ODS in mammalian burn wound infections, influencing both skin colonization and the subsequent dissemination of bacteria to internal organs.

### ODS interferes with nitric oxide metabolism in P. aeruginosa.

We previously characterized the ODS regulon using RNA seq ^3^. One previously unexplored finding was that the ODS regulon included the genes encoding NorB and NorC, two nitric oxide reductases. It is well-established that nitric oxide (NO) has the capacity to induce motility and foster biofilm dispersal in *P. aeruginosa* ^13,14^, thus prompting us to examine the intriguing interplay between these two signaling systems. Building upon this insight, we proceeded to further probe this relationship by deleting the norCB operon (*ΔnorCB*) and determining its effects on the ODS-dependent responses of *P. aeruginosa* to exogenous oleic acid.

**Figure 2.**
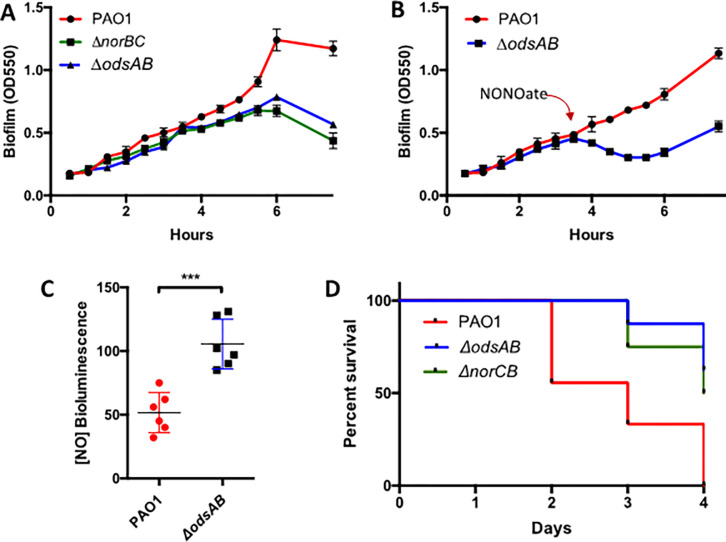
ODS regulates the levels of the nitric oxide (NO) signaling molecule. A) The time course of biofilm formation for PAO1, *ΔodsAB*, and *ΔnorCB* is depicted. Both *ΔodsAB* and *ΔnorCB* mutants exhibited a similar kinetic pattern of biofilm formation, suggesting a functional linkage between these metabolic pathways. B) The time course of biofilm dispersion post-treatment with the NO donor, NONOate, is illustrated. *ΔodsAB* displayed increased sensitivity to exogenous NO. NONOate (1 mM) was introduced at 3:45 hours after the initiation of the culture. The results shown in panel A and B are representative of three experiments. C) NO accumulation at higher concentrations in the supernatant of *ΔodsAB* compared to PAO1 WT is demonstrated. NO levels were measured by bioluminescence using an *E. coli* sensor strain ^27^. Each dot represents a biological replicate with statistical analyses performed using a two-tailed Student’s t-test (*** for P < 0.001). D) Kaplan Meier survival curve depicting infection-associated mortality for *ΔodsAB* and *ΔnorCB* mutants compared to PAO1. Mantel-Cox test was employed for statistical analysis of survival data.

Consistent with the connection between ODS and the NO signaling systems, both *ΔodsAB* and *ΔnorCB* strains exhibited a similar, diminished, pattern of biofilm formation over time *in vitro* (Fig. 2A). Furthermore, the *ΔodsAB* biofilm dispersed more rapidly than the PAO1 biofilm when exposed to the NO donor NONOate at a concentration of 1mM (Fig. 2B). Importantly, the notion that *ΔodsAB* experienced higher NO levels in culture compared to WT PAO1 were further supported by employing an *E. coli* bioluminescent NO biosensor, which consistently had greater bioluminescence when co-cultured with *ΔodsAB*, a result indicative of higher NO (Fig. 2C). Thus, activation of ODS, regulates the neutralization of NO via the production of NorB and NorC. With regard to *in vivo* experiments, our mouse burn model demonstrated an attenuated phenotype for the *ΔnorCB* mutant strain (Fig. 2D). Moreover, our observations did not reveal a significant difference between *ΔodsAB* and *ΔnorCB* mutants, which supports the notion that ODS functions, at least partially, through the regulation of NO levels *in vivo*.

### Immunized mice are protected from *P. aeruginosa* dissemination and death.

**Figure 3.**
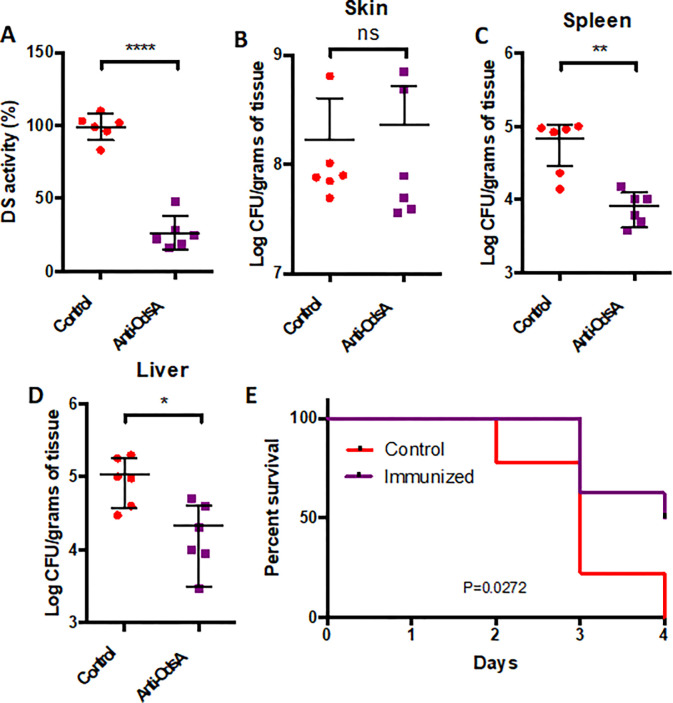
Immunized mice are protected from *P. aeruginosa* dissemination and death. A) Serum from mice immunized with OdsA demonstrated the ability to inhibit oxylipin production *in vitro*, indicating that OdsA-specific antibodies can effectively block OdsA activity. While no significant difference was observed in B) levels of skin colonization between OdsA-immunized mice and control mice, immunization with OdsA provided partial protection against bacterial dissemination from the skin to the C) spleen and D) liver. E) Kaplan Meier survival curves depict the significant benefit of OdsA immunization in enhancing survival rates. Non-immunized mice exhibited 100% mortality within four days post-infection, whereas OdsA-immunized mice showed a 50% survival rate during the same timeframe. Statistical analyses were conducted using Student’s t-test for panels A to D, while Mantel-Cox test was utilized for the survival data in panel E. The study was conducted with two independent experiments, each involving three mice per condition, totaling six mice for each group. Each dot denotes an individual mouse.

OdsA is released by *P. aeruginosa* into the extracellular milieu, where it catalyzes the conversion of oleic acid into oxylipins ^5^.This prompted us to explore OdsA as a potential target for a neutralizing antibody. Here, we overexpressed OdsA containing a histidine tag at its carboxy terminus and purified it using an immobilized metal affinity chromatography column (Fig S3A, B). Mice were immunized with recombinant OdsA, while a control group of mice received PBS. Upon completion of the immunization regimen, serum samples were assessed via immunoblotting to ascertain their ability to detect OdsA (Fig S3C). These confirmed anti-OdsA sera demonstrated the ability to block the conversion of oleic acid to oxylipins *in vitro* (Fig. 3). Subsequently, OdsA-immunized mice underwent burn injury and were infected with PAO1. Although no statistically significant difference was observed in terms of *P. aeruginosa* skin colonization between the OdsA-immunized mice and PBS-vaccinated control group (Fig. 3B), a reduction in the ability of the bacteria to disseminate became apparent. Specifically, OdsA-immunized mice had reduced PAO1 titers in both the spleen (Fig. 3C) and the liver (Fig. 3D) compared to the control mice. Most crucially, a mortality analysis indicated a significant protective effect of OdsA immunization, partially safeguarding burned mice from succumbing to mortality (Fig. 3E).

### An OdsA inhibitor prevented dissemination of P. aeruginosa.

**Figure 4.**
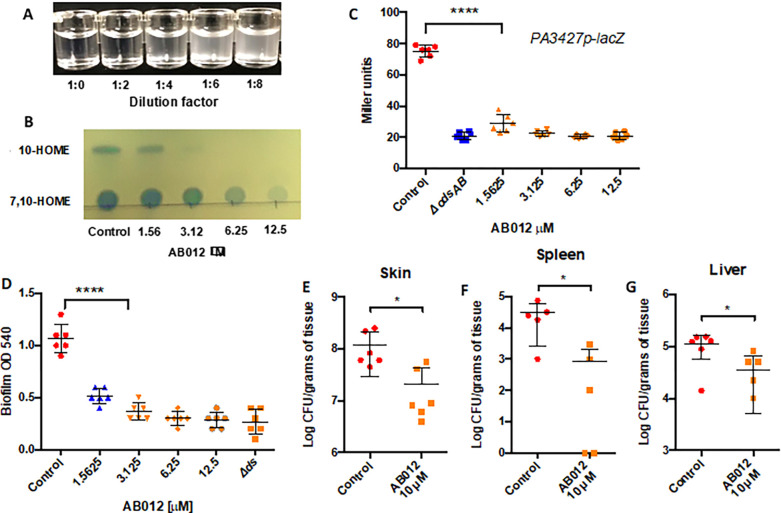
An OdsA inhibitor prevents dissemination of *P. aeruginosa*. A) Suspensions of 1 mg/mL oleic acid were treated with serial dilutions of a semi-purified fraction of oxylipin synthases, OdsA, and OdsB. The activity of oxylipin synthases rendered the media transparent in a concentration-dependent manner, attributed to the transformation of oleic acid into more hydrophilic oxylipin derivatives. B) Thin-layer chromatography (TLC) analysis of oxylipin products from a bioconversion with PAO1 supernatant and increasing concentrations of AB012 revealed inhibition of oxylipin synthase activity by AB012 *in vitro*. C) AB012 exhibited inhibition of lacZ expression under the control of the PA3427 promoter. PA3427 served as a reporter of ODS induction due to its high induction by oxylipins. D) AB012 inhibited biofilm formation in a concentration-dependent manner. E) Mice treated intradermally with AB012 demonstrated reduced skin colonization, along with decreased dissemination to F) the spleen and G) the liver. The study was conducted with two independent experiments, each involving three mice per condition, totaling six mice for each group. Each dot denotes an individual mouse. Statistical analyses were conducted using Mann-Whitney U-test for panels C and D, and Student’s t-test for panels E to G.

The presented results prompted us to develop a High-Throughput Screening (HTS) assay. We aimed to identify small molecules targeting oxylipin synthesis for potential therapeutic treatment of *P. aeruginosa* infections in burn lesions. Our screening strategy relied on the differential solubility of oleic acid, the diol-synthase pathway substrate, compared to the oxylipin products. Notably, a 10mM suspension of oleic acid, initially rendering a cloudy appearance, became clear when treated for one hour with a semi-purified fraction of the oxylipins synthases isolated from a free-cell supernatant of PAO1 (Fig. 4A). This difference in transparency facilitated easy monitoring of the conversion of oleic acid into oxylipins by measuring the optical density of the surrounding medium at a wavelength of 600 nm (OD600). Compounds inhibiting oxylipin production were identified in wells where the medium remained at a high OD. Using this strategy, a library of 200,000 compounds was screened at 10 μM. The most promising compound, AB012 (Fig S4) underwent retesting for inhibition of oxylipin synthesis *in vitro* utilizing the semi-purified fraction of oxylipin synthase enzymes employed during the initial screening process (Fig. 4B). Notably, AB012 effectively suppressed the production of both 10-HOME and 7,10-DiHOME *in vitro*, suggesting its potential to target either OdsA or both enzymes within the pathway. While AB012 exhibited no impact on *P. aeruginosa* growth when tested at 10 μM, it demonstrated an ability to inhibit the expression of PA3427gene at the same concentration (Fig. 4C). It is worth mentioning that while the function of the PA3427 product is unknown, it is among the most highly induced genes following exposure to oxylipins. Independently, AB012 exhibited a concentration-dependent inhibition of biofilm formation in PAO1 (Fig. 4D). To validate its efficacy *in vivo*, AB012 was evaluated using the mouse burn model. Burn mice infected with PAO1 were administered 10 μM of AB012 intradermally. Encouragingly, the treated mice exhibited reduced capacity of PAO1 to colonize the skin (Fig. 4E) and disseminate to the spleen and liver compared to the untreated mice (Fig. 4F and G). Overall, these results serve as a promising proof of concept for the targeting of OdsA as a therapeutic.

## Discussion

*P. aeruginosa* exhibits a remarkable capacity to initiate both acute and chronic infections, a trait attributed to its proficiency in perceiving and adjusting to dynamic host environments ^1^. Extensive investigations have addressed the adaptive mechanisms facilitating *P. aeruginosa* pathogenicity in burn wounds (^15–17^). However, the precise signals governing the genetic programs leading to this type of acute infection remain largely unexplored. This study was motivated by the observation that burn wounds frequently manifest *P. aeruginosa* infections characterized by heightened invasiveness. Our goal was to unravel the molecular underpinnings of *P. aeruginosa* hyper-virulence during burn-related infections, focusing on the role of oleic acid, a major component of human adipose tissue, released during skin burns.

Building upon previous research, we sought to elucidate the involvement of the ODS regulatory system in virulence using a mouse burn model. Our findings revealed that the burn microenvironment triggers *P. aeruginosa* virulence through the release of oleic acid and in turn activation of the ODS regulatory system. Significantly, ODS activation not only promoted skin colonization, but also played a crucial role in facilitating bacterial translocation to deep tissue causing dissemination to internal organs, a hallmark of severe infections. The notion that the burn microenvironment is conducive to ODS activation due to the presence of free oleic acid is substantiated by the detection of ODS-derived oxylipins in burn lesions infected with the PAO1 WT strain, contrasting with their absence in lesions infected with the ODS-deficient mutant. Furthermore, the specificity of ODS in promoting *P. aeruginosa* virulence in a burn context was underscored by the lack of discernible differences between PAO1 and the *ΔodsAB* mutant in the skin of otherwise healthy mice. It is noteworthy that oleic acid typically is absent in the airway or lungs, as its presence can led to injury and inflammation ^18^. This helps to explain why *P. aeruginosa* infections in context of cystic fibrosis or chronic airway diseases are typically not immediately life-threatening.

Notably, our investigations have unveiled a compelling distinction between the WT strain and the *ΔodsAB* mutant, with the former exhibiting a significantly enhanced migration into the skin. This suggests an important role for the ODS system in fostering dissemination through the induction of skin internalization. Our experiments with confluent endothelial cell monolayers performed *in vitro* indicate this was not simply the result of differences in bacterial burden. Along such lines, the choice of vascular endothelial cell layers is particularly relevant, considering that *P. aeruginosa* must traverse this specific cell type to access blood vessels. Previous reports from our research highlighted the inhibitory effect of ODS on flagella-dependent motility (swimming and swarming) while concurrently inducing Type-4 pili-dependent motility, known as twitching. Based on our most recent results, we now hypothesize that this specific ODS-dependent phenotype may facilitate adhesion to biotic surfaces, promoting subsequent internalization into host tissues. Consistent with this hypothesis, existing research underscores the importance of Type IV pili in *P. aeruginosa’s* adherence to epithelial cells. Moreover, there is supporting evidence indicating the involvement of twitching motility in the translocation of corneal epithelial cell multilayers by *P. aeruginosa*, demonstrated both *in vitro* and *in vivo* ^19^. While our experiments do not pinpoint the precise mechanism through which ODS facilitates cell layer translocation, it unequivocally emphasizes the significance of this system in orchestrating the migration of *P. aeruginosa* within host tissues. Finally, we cannot overlook the potential direct effect of ODS-derived oxylipins on host defenses, potentially aiding in *P. aeruginosa* dissemination. Recent findings have reported the presence of ODS-derived oxylipin, 10-HOME, in women with infected breast implants. In this instance, 10-HOME was shown to polarize CD4 + T cells to the Th1 subtype *in vitro* and in mice, suggesting a possible immunomodulatory role in facilitating *P. aeruginosa* dissemination ^20^.

Our previous RNAseq analysis revealed that ODS activation positively regulates the expression of nitric oxide reductase and decrease the expression of NO synthase compared to the wild type ^3^, our current investigation established that NO accumulates at substantially higher levels in the supernatant of *ΔodsAB* compared to the wild type *in vitro*. While NO is recognized for inducing biofilm dispersion in *P. aeruginosa* at nanomolar concentrations, its role during acute infections, such as burn-related infections, remains unknown. Our hypothesis posits that ODS-mediated reduction of NO induces tissue invasion through the induction of twitching motility. However, given the intricate nature of the NO network, we acknowledge the possibility of other NO-dependent mechanisms having an important role. Remarkably, our findings suggest that *P. aeruginosa* also has the capability to deplete exogenously produced NO. Consequently, we propose a potential role for ODS in scavenging host-produced NO and that this may restrict blood vessel dilation at the burn site, inhibiting immune cell infiltration and wound healing, both an additional mechanism by which ODS might promote bacterial persistence and opportunity for penetration of the subdermis.

Encouragingly, our results demonstrated that a polyclonal antibody targeting OdsA effectively blocks ODS activity *in vitro*, and that mice immunized with OdsA showed partial protection against disseminated infection following burn injury. Our ongoing efforts will focus on the development of a monoclonal anti-OdsA for potential therapeutic interventions. Our findings also prompted us to develop a strategy to identify small molecules able to block the ODS pathway. Drugs targeting enzymes involved in oxylipin production in mammals and fungi, such as aspirin, diclofenac, ibuprofen, and imidazole derivatives, have been extensively commercialized. However, the role of oxylipins in bacterial pathogenesis has received less attention, and currently, there are no commercially available drugs specifically designed to block bacterial oxylipin synthesis. The HTS assay identified several promising candidates that efficiently block oxylipin synthesis *in vitro*. As a proof of concept, we tested one of the most promising compounds for its ability to interfere with *P. aeruginosa’s* capability to disseminate from the skin to internal organs. As anticipated, this compound significantly impacted *P. aeruginosa’s* ability to disseminate to internal organs. While the selected compound requires further optimization, these results showcase the potential of targeting the ODS system for intervention during burn lesion infections.

In summary, our study significantly advances our comprehension of the nuanced interplay between environmental cues and virulence in *P. aeruginosa*. We establish free oleic acid as a discernible burn marker recognized by *P. aeruginosa* through the ODS system, triggering a cascade of events that promote virulence. This study represents a groundbreaking discovery, uncovering the role of oxylipins, produced by prokaryotes, akin to those seen in other pathogens like fungi, as pivotal signaling molecules in bacterial interactions with mammalian hosts ^21^. Specifically, these findings shed light on the intricate relationship between ODS, NO, and the pathogenicity of *P. aeruginosa*, particularly in the context of heightened virulence during burn wound infections. Moreover, our research highlights a promising avenue for therapeutic intervention aimed at mitigating dissemination following infection.

## Methods

### Strains.

*Pseudomonas aeruginosa* strain PAO1, sourced from the Manoil Lab at the University of Washington in Seattle, WA, USA, served as the parental strain throughout our investigation. The isogenic mutant *ΔodsAB* (diol synthase operon deletion mutant) was obtained following previously established protocols ^6^. The construction of *ΔnorCB* (*norCB* operon deletion mutant) involved allelic exchange utilizing the suicide vector pEX100Tlink, which contained the upstream and downstream regions of the *norCB* operons (pEX*ΔnorCB*). This vector facilitated an in-frame deletion of the *norCB* operon within the PAO1 chromosome. PCR and sequencing were employed to verify the mutant genotype. To complement the mutant, the mutated allele was replaced with the original copy from the parental strain PAO1, again employing allelic exchange. Green fluorescent *P. aeruginosa* strains were generated through transformation with plasmids pMF230, which constitutively express GFP. Plasmids pMF230 (Addgene plasmids #62546), generously provided by Michael Franklin of Montana State University, were utilized for this purpose. *Escherichia coli* DH5α (Invitrogen) served as the host for plasmid constructions, while *E. coli* S17–1 λpir, a gift from Jorge Benitez of Morehouse School of Medicine, was utilized as a donor strain for bacterial conjugation when necessary.

### Culture conditions

The strains were routinely cultivated in lysogeny broth (LB) medium at 30 °C, with agar incorporated when solid medium was necessary. To segregate the suicide plasmid from merodiploids during the construction of *ΔnorCB* by allelic exchange, LB agar devoid of NaCl but supplemented with 15% sucrose was employed. For biofilm formation assays, M63 medium was utilized, supplemented with 2% glucose, 5% casamino acids, and 1 mM MgSO_4_ (referred to as M63 complete). Antibiotics were supplemented as needed, with ampicillin (Amp) at 100 μg ml^−1^ and carbenicillin (Cb) at 300 μg ml^−1^. To induce oxylipin production and purification, cultures were supplemented with 90% oleic acid (Sigma 364525). When investigating biofilm formation *in vitro*, M63 complete media was supplemented with either 99% oleic acid (Sigma O1008) or purified oxylipins as required.

### Thin layer chromatography

Thin layer chromatography (TLC) experiments were conducted using Whatman silica gel plates (60 Å), measuring 20 × 10 cm with a thickness of 200 μm. The mobile phase consisted of a mixture of hexane, ether, and acetic acid in proportions of 80:20:5, respectively. Visualization of the separated compounds on the TLC plates was achieved by treating them with a solution of 10% phosphomolybdic acid in ethanol.

### Purification of 10-HOME and 7,10-DiHOME oxylipins.

The supernatant from a 500 ml PAO1 culture cultivated in M63 complete medium supplemented with 1% oleic acid was utilized for the purification of oxylipins produced through diol synthase activity. Following centrifugation of the culture at 8000 × g for 15 minutes, the supernatant was carefully retrieved and acidified to a pH of 2 using hydrochloric acid. Subsequently, a one-to-one volume ratio organic extraction was conducted employing ethyl acetate. The organic phase was then evaporated, yielding a dried mixture that was dissolved in 3 ml of ethyl acetate for further purification steps. Purification of the oxylipins was carried out utilizing an Isco Teledyne Combiflash Rf 200 equipped with four channels and a 340CF ELSD (evaporative light scattering detector). Pre-packed cartridges of Universal RediSep solid sample loading (5.0 g silica) were employed for crude product absorption, followed by purification on 24 g silica RediSep Rf Gold Silica columns (20–40 μm spherical silica) using an ascending gradient of ethyl acetate (solvent B) against hexane (solvent A). Fractions corresponding to each detected peak were pooled and evaporated before being dissolved in ethanol. The purity of the oxylipins was assessed through HPLC/MS analysis.

### HPLC/MS analysis.

Mass spectrometry analysis was performed as described previously ^22^. Purified 7,10 Di-HOME and 10-HOME were prepared as stock solutions at a concentration of 1 mg ml−1 in ethanol. From these stock solutions, samples for analysis were prepared by diluting in ddH_2_O containing 0.1% formic acid. Each sample, with a 20 μl injection volume, was loaded onto a Synergi Hydro-RP 80A 250 × 2 mm C18 column (Phenomenex), employing a Shimadzu Prominence System Binary Pump (Shimadzu Scientific Instruments, Inc., Columbia, MD, USA) at a flow rate of 350 μl min−1. Mobile phase A consisted of ddH_2_O with 0.1% formic acid, while mobile phase B comprised acetonitrile with 0.1% formic acid. The gradient elution started at 10% B and increased to 80% B over 11 min, followed by a ramp to 100% B at 14 min, then re-equilibrated to initial conditions over 6 min, resulting in a total runtime of 20 min per analysis. The SCIEX 4000 Triple Quadrupole Mass Spectrometer (Concord, Ontario, Canada) operated in ESI negative ion mode, with nitrogen serving as the nebulizer and curtain gas (CUR=20). Collision gas, collision energy, and temperature were set at 10 °C (−30 eV for 10-HOME, −34 eV for 7,10-DiHOME) and 600 °C, respectively. Gas settings GS1 and GS2 were maintained at 40 °C and 60 °C, respectively. Analyst 1.6.2 software controlled the LC-MS/MS system.

### Quantification of biofilm formation.

Biofilm assays were conducted in accordance with the O’Toole protocol ^23^. Initially, *P. aeruginosa* strains were cultured overnight on LB agar plates at 37 °C. Bacterial suspensions were then prepared in M63 medium to achieve an OD600=1. Subsequently, 10 microliters of the bacterial suspension were inoculated into each well of a 96-well microtiter plate containing 200 μl of M63 complete media. When necessary, oleic acid or pure oxylipins were supplemented to the medium at desired concentrations. Biofilms were allowed to develop overnight at 30 °C. For quantification of biofilms, the wells were washed twice with 1 × PBS, and then 200 μl of 0.1% crystal violet was added to each well, followed by a 10-minute incubation period. Afterward, the wells were washed three times with 1 × PBS, and the crystal violet-stained biofilm was solubilized with 250 μl of 30% acetic acid. Absorbance was measured at 550 nm to determine biofilm formation.

### HTS assay.

The primary assay utilized in this study focused on the differential solubility properties of the diol-synthase pathway substrate, OA, compared to its oxylipin products. A suspension of OA at a concentration of 1 mg/mL results in a cloudy solution due to the formation of micelles. Upon treatment with semipurified diol-synthase enzymes, the suspension becomes transparent, indicating the conversion of OA to more soluble oxylipins. To identify inhibitors of diol-synthase activity, compounds were tested for their ability to maintain the cloudiness of the OA suspension, as this would suggest inhibition of the enzyme. The cloudiness of the suspension was quantified by measuring its optical density at a wavelength of 600 nm (OD600). A clear suspension after treatment would indicate enzyme activity, while a cloudy suspension would signal inhibition. The assay was validated in a 384-well plate format and subsequently adapted for robotic automation. The HTS group at SR conducted a screen of 200,000 compounds at a concentration of 10 μM, with each compound being tested in duplicate to ensure reproducibility and accuracy. Positive hits from the initial screen were further analyzed through a 10-point dose-response assay to determine their IC50 values. One particularly promising hit was selected in this study as a proof-of-concept for the feasibility of ODS inhibition *in vivo*.

### Trans-well assay.

Trans-well experiments were performed as described previously^24^. Initially, 5.0 × 10^5 murine colon epithelial cells (MCEC) were seeded onto Trans-well permeable inserts (12 mm diameter, 3-μm pore size; Costar) in 12-well plates and cultured for a minimum of 48 hours at 37°C with 5% CO2. Subsequently, 5.0 × 10^5 colony-forming units (cfu) of *P. aeruginosa* were introduced to the cells, followed by centrifugation at 500 × g for 5 minutes and incubation for 30 minutes at 37°C with 5% CO2. Following this, the inserts were washed thrice with prewarmed phosphate-buffered saline (PBS) and transferred to new plates, followed by incubation for 1 hour in Dulbecco’s Modified Eagle Medium (DMEM). The translocated bacteria were quantified by enumerating the colony-forming units (CFU) recovered in the lower chamber.

### Virulence assay in mouse burn model.

Mice infection was conducted following previously established protocols ^25^. In summary, five- to six-week-old BALBc mice (Jackson Labs) were anesthetized using a mixture of xylazine and ketamine. Their dorsal region was prepared by shaving with an electric clipper followed by depilation using depilatory cream. A thermal burn was then induced using a hot bar ^26^, after which intradermal infection was administered using 100 μl of bacterial suspension containing 10^5^ CFU (colony-forming units) of either *P. aeruginosa* PAO1 or the *odsAB* mutant strain. Subsequent to infection, the bacterial burden in the skin, liver, and spleen was evaluated. This involved homogenizing the respective organs and plating serial dilutions onto LB agar plates for quantification.

### *P. aeruginosa* imaging inside mice skin.

Mice infected with 10^5 colony-forming units (cfu) of PAO1 or *ΔodsAB* constitutively expressing GFP were euthanized 24 hours post-infection, and a skin biopsy was embedded in Optimal Cutting Temperature Compound (Tissue-Tek, 4583) and frozen until analysis. Visualization of bacteria within the mice skin was performed using the Leica LMD 6 and Nikon Eclipse Ti microscope.

### Treatment of burn wound infection with an oxylipin synthase inhibitor.

One hour post-infection, mice were subjected to intradermal treatment with either 100 μl of a 10 micromolar solution of AB012 or 100 μl of phosphate-buffered saline for control purposes. Following treatment, mice were monitored daily for symptoms and mortality over a span of 10 days. Alternatively, mice were euthanized at either 24 or 48 hours post-infection to assess bacterial load in the skin, liver, and spleen.

### OdsA expression and mice immunization.

The OdsA gene was amplified using the PAO1 chromosome as a template with specific primers. The resulting fragment was then cloned into the PET23a expression vector, incorporating a His tag. This construct was subsequently transformed into *E. coli* BL21 (DE3) for protein expression. Upon reaching an OD600 of 0.4 to 0.6, cultures were induced using 1 mM isopropyl-β-d-thiogalactopyranoside (IPTG) for 4 hours at 37°C on a shaker. Bacterial cells were harvested by centrifugation at 4,000 × g for 15 minutes. The resulting cell pellets were resuspended in buffer A (50 mM Tris-HCl [pH 7.5] and 150 mM NaCl) containing 1 mM phenylmethylsulfonyl fluoride (PMSF), a serine protease inhibitor, and then sonicated at 35% amplitude (2 s on/2 s off) for 30 minutes on ice for lysis. Subsequently, the lysate was centrifuged at 12,000 × g for 30 minutes at 4°C. The overexpressed protein present in the supernatant was purified using a cobalt resin column following the manufacturer’s instructions for His tag purification. For immunization studies, mice were subcutaneously administered 0.1 mg of OdsA on day 1, followed by a booster of 0.05 mg of the protein administered 14 days later. Peripheral blood samples for serum collection were obtained one week post-booster via retro-orbital bleeding of anesthetized mice just before euthanasia.

### Detection of oxylipins in the skin of *P. aeruginosa* infected mice.

Groups of three mice were subjected to thermal burns and subsequently infected either with PAO1 or a *odsAB* deficient mutant. After 24 hours, the mice were euthanized, and their skin was homogenized using an Omni THQ homogenizer equipped with disposable Omni Tips plastic generator probes (OMNI international) in 2 ml of PBS 1×. The homogenates underwent centrifugation to remove tissue and bacterial debris, following which total fatty acids were extracted according to the method outlined previously (refer to the section titled “Purification of diol synthase-derived oxylipins”). Extracted samples were then subjected to analysis using HPLC/MS (refer to preceding sections for TLC and HPLC/MS analyses) to detect the presence of 10-HOME and 7,10-DiHOME. The identification of oxylipins was carried out utilizing the Multiple Reaction Monitoring (MRM) method, with mass transitions m/z 297.3/155.1 for 10-HOME and 313.3/141.1 for 7,10-DiHOME.

### Ethics statement.

The animal experimental design was approved by the Institutional Animal Care and Use Committee at The University of Alabama at Birmingham, UAB (protocol no. IACUC-22197).

### Statistical analysis.

TThe survival data from mice experiments were visualized through Kaplan-Meier plots, and their comparability was assessed via the log-rank (Mantel-Cox) test. Each experimental condition involved 6 mice. Subsequent analyses utilized either one-way ANOVA or unpaired t-tests as appropriate. Statistical computations were conducted using GraphPad Prism 8 software (GraphPad Software, La Jolla, CA).

## Data Availability

The authors affirm that the data underpinning the conclusions of this study can be found within the article itself, along with its supplementary information files, or can be obtained directly from the corresponding author upon request.

## Significance Statement

Sepsis stemming from *Pseudomonas aeruginosa* burn wound infection is a primary cause of mortality in burn trauma patients. Herein, we show that *P. aeruginosa* utilizes host-derived oleic acid, released from adipose tissue, to activate the oxylipin-dependent quorum-sensing system (ODS). ODS in turn regulates levels of nitric oxide, a pivotal signaling molecule within bacteria, and this facilitates invasion of burned skin and systemic spread. Inhibition of oxylipin generation, either with antibody against the responsible enzyme or with a pharmacological agent, protected mice from lethal infection post-burn. Our results provide a molecular explanation for the hypervirulent phenotype of *P. aeruginosa* within burn infections and informs on novel treatments to prevent disseminated infection and enhance their recovery prospects.
